# A Dominant X-Linked QTL Regulating Pubertal Timing in Mice Found by Whole Genome Scanning and Modified Interval-Specific Congenic Strain Analysis

**DOI:** 10.1371/journal.pone.0003021

**Published:** 2008-08-22

**Authors:** Wangsheng Zhu, Zhongpeng Fan, Chao Zhang, Zhengxia Guo, Ying Zhao, Yuxun Zhou, Kai Li, Zhenghong Xing, Guoqiang Chen, Yinming Liang, Li Jin, Junhua Xiao

**Affiliations:** 1 College of Chemistry, Chemical Engineering & Biotechnology, Donghua University, Shanghai Songjiang, People's Republic of China; 2 Joint Laboratory for Model Animal Biodiversity, Shanghai Pudong, People's Republic of China; 3 Shanghai British SIPPR/BK Lab Animal Ltd, Shanghai, People's Republic of China; 4 School of Life Science, Fudan University, Shanghai, People's Republic of China; Centre National de la Recherche Scientifique, France

## Abstract

**Background:**

Pubertal timing in mammals is triggered by reactivation of the hypothalamic-pituitary-gonadal (HPG) axis and modulated by both genetic and environmental factors. Strain-dependent differences in vaginal opening among inbred mouse strains suggest that genetic background contribute significantly to the puberty timing, although the exact mechanism remains unknown.

**Methodology/Principal Findings:**

We performed a genome-wide scanning for linkage in reciprocal crosses between two strains, C3H/HeJ (C3H) and C57BL6/J (B6), which differed significantly in the pubertal timing. Vaginal opening (VO) was used to characterize pubertal timing in female mice, and the age at VO of all female mice (two parental strains, F1 and F2 progeny) was recorded. A genome-wide search was performed in 260 phenotypically extreme F2 mice out of 464 female progeny of the F1 intercrosses to identify quantitative trait loci (QTLs) controlling this trait. A QTL significantly associated was mapped to the DXMit166 marker (15.5 cM, LOD = 3.86, p<0.01) in the reciprocal cross population (C3HB6F2). This QTL contributed 2.1 days to the timing of VO, which accounted for 32.31% of the difference between the original strains. Further study showed that the QTL was B6-dominant and explained 10.5% of variation to this trait with a power of 99.4% at an alpha level of 0.05.The location of the significant ChrX QTL found by genome scanning was then fine-mapped to a region of ∼2.5 cM between marker DXMit68 and rs29053133 by generating and phenotyping a panel of 10 modified interval-specific congenic strains (mISCSs).

**Conclusions/Significance:**

Such findings in our study lay a foundation for positional cloning of genes regulating the timing of puberty, and also reveal the fact that chromosome X (the sex chromosome) does carry gene(s) which take part in the regulative pathway of the pubertal timing in mice.

## Introduction

Puberty is an important and complex biological process that involves sexual development, accelerated linear growth, and adrenal maturation. The maturation of the hypothalamic-pituitary-gonadal (HPG) axis underlies the development of puberty in mammalians [1]–[4]. Environmental and metabolic factors are important regulators of the neuroendocrine axis that affects growth and development of puberty; however these influences are superimposed upon substantial genetic control [5].

In mice, genetic influences on the timing of pubertal events such as age at vaginal opening (VO), first vaginal cornification and the onset of cyclicity, have been extensively studied [6], [7]. Though the genetic factors specifying the timing of vaginal opening and first vaginal cornification differ from those regulating the onset of cyclicity, the three pubertal events are all genetic characteristics of the timing of puberty [7]. Owing to its easy-manipulation, vaginal opening is widely used as assessment to characterize the timing of puberty in rodents [8]–[10].

The studies on model animals have provided much information about how genetic factors regulate the timing of puberty in mammals. Using a panel of chromosome substitution strains [CSSs between inbred strains C57BL/6J (B6) and A/J], researchers found that chromosome 6 and 13 might harbor gene(s) regulating the timing of VO [9]. Subsequent linkage analysis and phenotyping of 12 congenic strains between the two strains mapped at the distal end of chromosome 6 a Quantitative Trait Locus (QTL) responsible for the regulation of the pubertal timing in mice [10]. It is noteworthy that none of the published data have provided evidence supporting the association between the sex chromosome gene(s) and the timing of puberty. However, other investigations on human Xq have suggested its potential regulatory roles in the pubertal timing [11]–[13]. Sex chromosome in mammals is the genetic basis of the discrepancy between two sexes and gene(s) on it can directly affect brain sexual differentiation [14]. In most mammals, the timing of puberty is sex and also species dependent, for example, puberty in girls is triggered earlier than boys but male lambs begin puberty before female lambs [15]. In this work, we provided the first direct evidences that chromosme X harbor gene(s) regulating puberty timing in mice.

In this study, QTL analysis was performed in reciprocal pedigrees originated from C3H/HeJ (C3H) and C57BL/6J (B6) inbred mice. The two inbred strains were investigated in our study because the timing of VO differed significantly from each other (*P*<0.05) and the discrepancy was ascribed to genetic factors [6]. Our previous study showed that the heritability of the timing of VO in direct and reciprocal crosses between B6 and C3H inbred female mice is 68.51% and 63.97% respectively [16]. In this study, the population generated from B6 female and C3H male were defined as the direct cross and the opposite as the reciprocal cross. Pedigrees studied were from three generations: inbred progenitors(B6, C3H); F1 crosses (B6♀×C3H♂[B6C3HF1], C3H♀×B6♂[C3HB6F1]); and F2 crosses (B6C3HF1♀×B6C3HF1♂[B6C3HF2], C3HB6F1♀×C3HB6F1♂[C3HB6F2]). Whole genome scanning was performed in 260 phenotypically extreme F2 mice out of 464 female progeny of the F1 intercrosses. Additionally, one modified chromosome substitute strain (mCSS) and a series of modified interval-specific congenic strains (mISCSs) were constructed through backcrossing F1 hybrids to parental strain C3H. Nomenclaturally, a congenic strain is formed by backcrossing a locus of interest into an inbred mouse strain for 10 or more generations. And the mISCSs here refer to the congenic strains produced with less generations of backcrossing, in order to be time and fund effective for this study. Analysis of the congenic strains were used to verify and narrow the chromosome regions that were significantly associated with the pubertal timing in the genome scanning. Finally, the first significant QTL regulating the timing of puberty on mice chromosome X was identified.

## Results

### Genetic variation in timing of VO among mice in each generation

The distributions of age at vaginal opening in three pedigrees including parental strains, F1 hybrids and F2 hybrids were reported to be normal in our previous study [16]. The data showed that the heritability of the age at vaginal opening in direct and reciprocal crosses between B6 and C3H inbred mice is 68.51% and 63.97% respectively.

### Identification of QTL genes in three F2 populations

**Figure 1:**
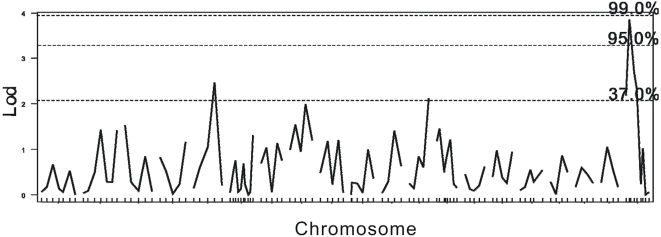
Linkage data for age at VO in the reciprocal cross of F2 population (C3HB6F2). The horizontal axes indicate chromosomal locations and marker order between markers. Dashed lines indicate genome-wide suggestive (*P*<0.63), empirical significant (*P*<0.05), and (*P*<0.01), LOD statistics at 2.03, 3.27, and 3.93, respectively.

**Figure 2:**
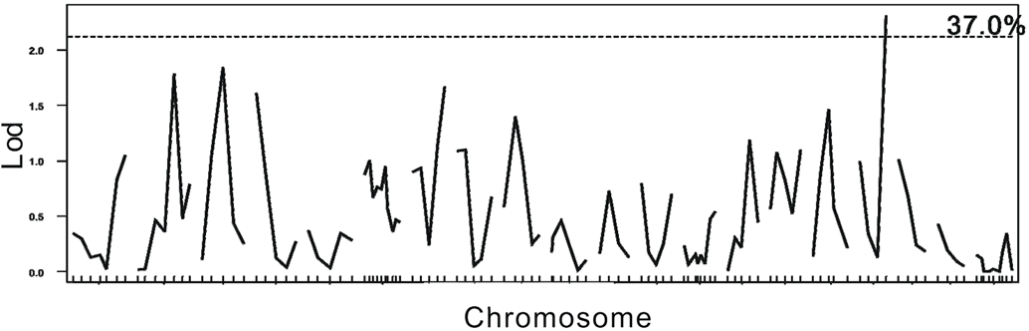
Linkage data for age at VO in the direct cross of F2 population (B6C3HF2). The horizontal axes indicate chromosomal locations and marker order between markers. No positive locus was investigated in this population.

**Figure 3:**
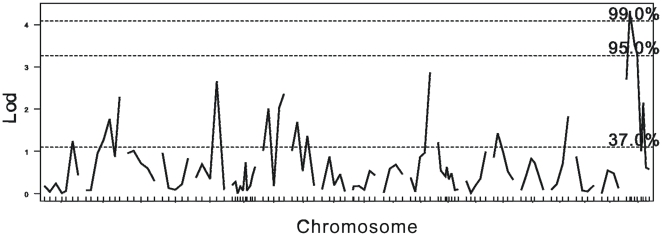
Linkage data for age at VO in the mixture F2 population. The horizontal axes indicate chromosomal locations and marker order between markers. LOD scores of 2.00, 3.26, 3.95 are established as the cut-offs for statistical significance (p<0.63, p<0.05 and p<0.01 respectively).

First, a genome wide permutation was performed to generate threshold. According to the genome wide permutation delivered threshold, three QTLs associated with the timing of VO were identified in the C3HB6F2 population (Figure 1): the one on chromosome X was empirically significant (DXMit166, 15.5 cM, LOD = 3.86, p<0.05), and the other two on chromosome 5 and 12 were only slightly suggestive (D5Mit367, LOD = 2.47; D12Mit144, LOD = 2.13, p>0.1). And only a suggestive QTL on chromosome 17 was identified (D17Mit129, LOD = 2.314, p>0.1) within the B6C3HF2 population, but the significant DXMit166 marker mapped to chromosome X in C3HB6F2 population was not present in this population (Figure 2). We further analyzed the mixture population (grouping the C3B6F2 and B6C3HF2 populations) and found the DXMit166 was empirically highly significant (LOD = 4.33, p<0.01) and no significant loci was found on autosomes (Figure 3).

Furthermore, to make a more reliable threshold, a stratified permutation delivered threshold was introduced. Also, considering the specified genetic model of chromosome X, the permutation performance of autosome and chromosome X were specified (Table 1). According to the stratified and chromosome X specified permutation-derived threshold, the QTL located on chromosome X was still significant in both C3HB6F2 and the mixture population (p<0.01), but not in B6C3F2 population.

**Table 1:** LOD thresholds derived from stratified and chromosome X specified permutation.

	LOD thresholds derived from stratified permutation^1^					
	C3HB6F2		B6C3HF2		Mixture population	
P-value	Autosome	Chr X	Autosome	Chr X	Autosome	Chr X
0.01	4.03	3.50	4.05	3.66	4.25	3.77
0.05	3.32	2.85	3.43	3.09	3.36	2.96
0.63	2.04	1.58	2.07	1.70	2.01	1.55

1 : in this study, only some of mice were genotyped, so a stratified permutation was applied to determine the LOD thresholds. Also, the autosome and chromosome X were treated discriminately in permutation performing so as to generate a more reliable permutation derived threshold. The analysis was performed with the package R/QTL.

A power analysis was performed, DXMit166 was responsible for 10.5% of the total variance with 99.4% power at an alpha level of 0.05. And this locus contributed 2.1 days to the timing of VO, which accounted for 32.31% of total difference between the original parental strains.

### Genetic inheritance pattern of the DXMit166 Locus on ChrX

**Figure 4:**
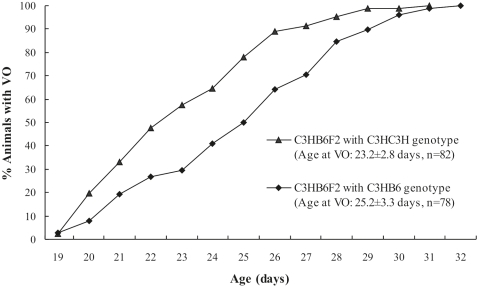
Comparison of the Timing of Vaginal Opening between different genotypes of DXMit166. The cumulative percentage of animals with vaginal opening (VO) is displayed. The distribution of age at VO of mice with different genotype at the peak linkage marker is shown.

To detect the genetic pattern of the significant QTL near DXMit166, the distribution of the age at VO of mice with different genotypes on the identified markers close to the LOD score peak, was compared within C3HB6F2 and B6C3HF2 populations. Because the QTL fell on chromosome X, the genetic pattern of it was in many ways like a backcross rather than F2. There are two genotypes for every marker in each population: C3HB6 and C3HC3H in C3HB6F2, C3HB6 and B6B6 in B6C3HF2. We found that the distribution of age at VO with different genotype varied significantly (p<0.0001) in the C3HB6F2 population (Figure 4). As significant difference has not been observed between genotypes of B6B6 and C3HB6 in B6C3HF2 mice population (p = 0.955, data not shown), it can be inferred that the effect of genes near DXMit166 of B6 regulating the timing of VO is dominant.

To verify the dominant nature of the ChrX QTL, mCSS mice with heterozygous chromosome X (referred to as C3HC3H-X^B^X^C^) and the C3H inbred mice were studied. The onset of VO in C3H-X^B^ X^C^ mice was significantly later than that of the progenitor C3H strain (P<0.001). The later onset of VO in the C3H-X^B^X^C^ population further supports that the regulatory effect of QTLs mapped to chromosome X on timing of VO is dominant. (Table 2)

**Table 2:** Comparison of the timing of vaginal opening and body weight at VO between C3H-X^B^ X^C^ CSS and progenitor C3H strain.

Genotype (marker DXMit166)	Number of mice (No. of Litters)	Age at VO in days (mean±SD)	Body weight at VO (g) (mean±SD)
C3HC3H	28(10)	22.1±1.2^1^	10.73±1.2^2^
C3HC3H -X^B^X^C^	15(6)	23.7±1.9	10.72±1.6

The age at onset of VO of C3HC3H was significantly earlier than that of C3HC3H -X^B^X^C^ (^1^: p<0.0001), but the body-weight at VO was not statistically different between the two strains (^2^: p = 0.955). (VO, Vaginal Opening; SD, Standard Deviation; C3HC3H -X^B^X^C^ refers to C3H gene background with a B6 X-chromosome.)

### Fine mapping of the candidate chromosome region on ChrX

**Figure 5:**
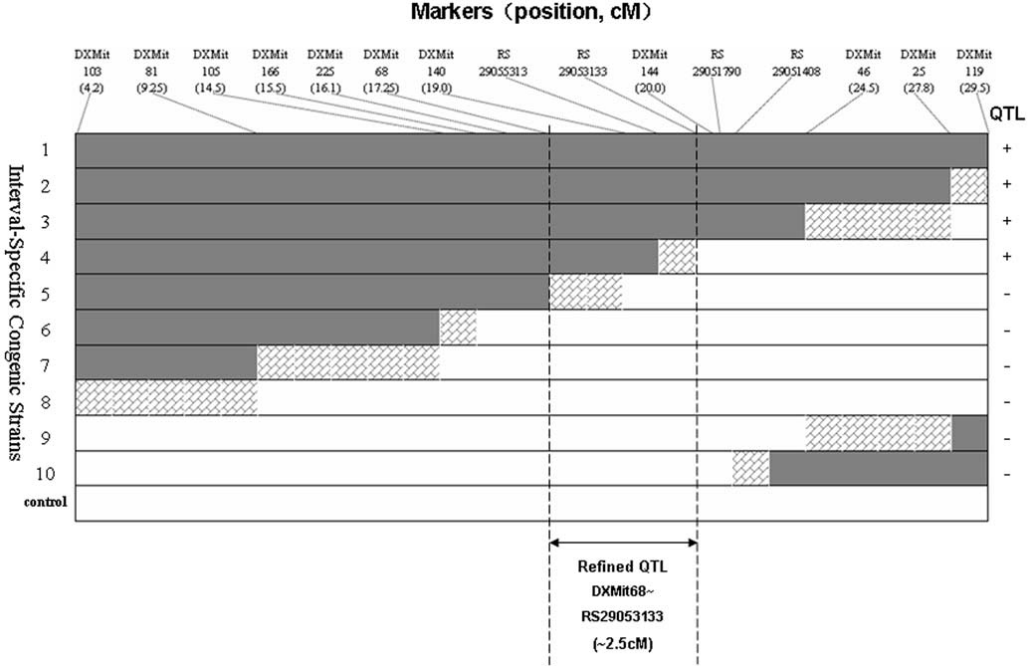
Interval-specific Congenic Strains. The genotype on chromosome X for each of the 10 congenic strains is represented by the horizontal bars. Shaded portions indicate a known homozygous B6 segment, unshaded portions represent a known homozygous C3H segment, and hatched regions depict an area where a crossover between C3H and B6 occurred. The QTL mapped from the F2 linkage analysis extends from dotted lines (17.25–20 cM). The boundary of the refined QTL is shown by the dashed line.

To confirm and refine the location of the QTL detected in whole genome scanning, ten mISCSs with intervals of interest on chromosome X of B6 inbred strain substituted into the C3H background (referred to as C3H-X^B^ X^C^) were developed and used to compare with the C3H inbred strain. The results of nonparametric tests showed that the strain 1∼4 mISCSs were significantly different from C3H inbred strain and were recorded as strains with QTL. The age at VO of strain 5∼10 was not significantly different from that of the C3H inbred strain, and such strains were absent of causative QTLs (Table 3). Based on the QTL information and allele distribution among those strains, a more narrowed interval of ∼2.5 cM between DXMit 68 and rs29053133 was identified (Figure 5).

**Table 3:** Comparison of the timing of vaginal opening and body weight at VO among ten ISCSs and the control strain C3H.

Strains ID*	Age at VO in days (mean±SD)	N. of Mice (N. of Litters)	Body Weight at VO (g) (mean±SD)
1	24.1±2.3^1^	21(6)	10.74±1.67
2	24.0±2.6^1^	18(5)	11.14±0.99
3	24.2±1.7^1^	23(8)	10.54±1.23
4	23.8±1.9^1^	28(9)	10.48±1.67
5	21.9±1.9^2^	34(10)	10.61±1.74
6	21.8±1.5^2^	17(6)	10.45±2.66
7	22.1±1.1^2^	21(4)	10.38±1.39
8	22.1±1.4^2^	13(4)	11.05±2.09
9	22.1±1.5^2^	27(8)	10.78±0.82
10	22.3±1.7^2^	17(6)	11.25±2.08
Control (C3H)	22.1±1.2	28(10)	10.73±1.2
Strains with QTL	24.0±1.97	90(26)	10.69±1.27
Strains without QTL	22.1±1.58	129(38)	10.72±1.56

ISCSs were generated by continually backcrossing F2 with C3H and the last progenies were from C3H mother. Age at VO of strains 1–4 is significantly different from the control strain C3H (^1^: p<0.0001), but strains 5–10 is not statistically different from C3H (^2^: p>0.5). SD, Standard Deviation.

### Association between body weight growth with age at VO

To study the association between body weight gain and age at VO in mice, the body weight at VO were compared between the strains with and without the QTL (Table 2, 3). First, body weights at VO did not differ between C3B6F2- X^C^X^C^ and C3B6F2- X^B^X^C^ mice at DXMit166 locus (p = 0.292, data not shown). In addition, based on the mCSS, we found that the age at VO in the C3HC3H –X^C^X^C^ was significantly earlier than that of C3HC3H -X^B^X^C^ (22.1±1.2 VS 23.7±1.9 days, p<0.0001), but the body-weight at VO was not statistically different between the two strains (p = 0.955)(Table 2). In other words, the strains C3HC3H-X^C^X^C^ grew faster than the strains C3HC3H -X^B^X^C^. Finally, we analyzed the mISCSs and also found that the body-weight at VO did not differ (p = 0.915) between congenic strains with (10.69±1.27 g) and without the QTL (10.69±1.27 g VS 10.71±1.56 g, p>0.5), although the age at VO was significantly different among them (24.0±1.97 days VS 22.1±1.58 days, p<0.00001). All of these results showed that different timing of VO among strains might have correlation with growth rate. The independent data from the experimental procedures described above lead to the postulation that the QTL causing VO variation is significantly associated with body weight.

## Discussion

Our previous data showed that the heritability of the timing of puberty in direct and reciprocal crosses between B6 and C3H inbred mice was 68.51% and 63.97%, respectively [16]. In this study, a QTL on chromosome X, which explained 0.5% of variation to this trait,was identified by whole genome scanning in two reciprocal pedigrees and was further confirmed and refined in mCSS and mISCS populations. Although it was found by chance, it is still very interesting in terms of the association determined between the sex chromosome and pubertal timing.

### Reciprocal F2 intercross design helps map X-linked QTLs regulating onset of VO

In this study, we used intercross (F2) from two inbred strains to examine the genetic factors regulating the timing of puberty in mice. Population design of the F2 intercross has been widely used in QTL searching experiments because it allows both additive and dominant QTL effects and epistatic interactions to be estimated [17]–[18]. However, the X-linked QTL analysis in female F2 population may be biased because only two genotypes can be investigated in a single direction of cross. In our study, the QTL on chromosome X would have been overlooked if only B6C3HF2 population had been investigated. The general rational explanation is that the mapped X-QTL was B6-dominant, and therefore, no phenotypic discrepancy between genotype of X^B^X^C^ and X^B^X^B^ could be noticed. In C3HB6F2 population, the QTL effect could be investigated because the population genotypes were X^B^X^C^ and X^C^X^C^. As a result, QTL searching in a F2 population derived from single direction cross could result in biased and false-negative conclusions. Accordingly the design of direct and reciprocal crosses can help to achieve a complete dissection of X-linked QTLs.

### Modified CSSs and ISCS were used to fine map the QTL on ChrX

Several strategies have been proposed to fine-map and narrow QTL intervals such as utilizing interval-specific congenic strains [19], genome-tagged mice [20], and advanced intercross lines [21]. Congenic strain construction was regarded as an efficient and reliable method in identifying the genes involved in many complex traits because such strains provide uniform genetic background in the study of a specific chromosome region [10], [22]. However, construction of congenic strains is time-consuming for the requirement of a number of repeated backcrosses. In our study, considering the strong genetic contribution of the QTL on ChrX, we selected ISCSs and CSS in the N_4_ population to refine the identified QTL. Theoretically, four generations of backcross mating result in 93.75% of genome of the recipient strain C3H which carries the introgressed chromosome region of interest from B6 [23]. The remnant B6 genetic background only accounts for 1/16 in N_4_ population, which is assumed to have little interference on the analysis of the QTL as a large number of samples studied. The average number of each mISCS used in our experiment was over twenty from more than four litters.

In the process of mISCS development, the male N3 hybrids were genotyped and the individuals with one recombination on chromosome X were selected to backcross with female C3H to generate female N4 hybrids. Because of the dominant effect of the QTL gene on ChrX, N4 hybrids were able to be used at fine mapping even though the mapped locus remained heterozygous. The dominant nature and experimental design were time and resource saving as additional backcrossing and intercrossing were not required.

In addition, such a method can avoid the possible maternal effects because the N_4_ hybrids are all generated from female C3H with the same gene backgrounds. In this study, the modified strategy was proved to be feasible in fine-mapping the QTL on chromosome X.

### The QTL Region on ChrX harbors genes regulating body-weight growth rate in mice

Our previous study and many other researches have suggested that the timing of puberty in mice is associated with body weight [16], [24]. In our study, we investigated that the body weight at VO was not significantly different between the mice with and without the QTL, though they attained the puberty onset at different time. This result indicated that pubertal timing was associated with growth rate in mice as, in general, the faster the mice grew, the earlier they attained puberty onset.

To study the nature of the identified QTL on chromosome X, all QTLs documented to date on the X chromosome were analyzed. Among all the 75 QTLs located on chromosome X, seven QTLs are body-weight related, three and four are obesity and postnatal growth rate related, respectively (http://www.informatics.jax.org/searches/marker_report.cgi). It is interesting that the body weight and postnatal growth rate related QTLs found in previous studies with C57BL/6J and wild mice *Mus.m.castaneus*, are concordant with the QTL region identified in our study [25]–[27]. The LOD peak of *castaneus* 10 week body weight QTL (*C10bw6*) positioned at 18.9 cM and postnatal body weight growth QTL (*Pbwg7*) located at 19 cM on chromosome X, both at the candidate region of our QTL that spanned from 17.25 to 20 cM. The QTL gene *Pbwg7* was reported to be male dependent because it was only found in male population and was absent in female mice. However, this finding is postulated to be biased as only one direct cross was performed in their experiments, in which B6 female mice were crossed to male wild mice. The X^B^X^W^ and X^B^X^B^ female populations were phenotyped however no positive result was obtained. It is possible that the QTL gene *Pbwg7* is B6-dominant. Interestingly, our study found that the QTL effect in the same chromosome region did have B6-dominant feature.

Other studies have also found that interval between 12.0 and 30.74 cM on chromosome X harbors gene(s) regulating mouse placenta development [28] which is widely regarded to program the offspring's prenatal and postnatal growth [29].

The concordance of QTLs for pubertal timing in mice with postnatal growth rate and prenatal placenta development indicated that the gene(s) on chromosome X regulating pubertal timing may be associated with prenatal and postnatal growth.

### Candidate Genes within the chromosome regions investigated

In candidate gene(s) discussion, the chromosome region under investigation is not restricted to the 2.5 cM but spans from DXMit103 (4.2 cM) to DXMit103 (29.5 cM). From current knowledge, the most likely candidate genes in that region are Pgrmc1, Gria3 and Plac1. One of the candidate genes, progesterone receptor membrane component 1(Pgrmc1), is a membrane-associated protein, which is required for progesterone to transduce its antiapoptotic action in gonad and is also expressed in anterior pituitary, both regarded as pivotal for the regulation of pubertal timing in mammalians [30]. Glutamate receptor, ionotropic, AMPA3 (alpha 3) (Gria3) is another candidate gene presented in the hypothalamus and its activation is stimulatory to LHRH/LH secretion which results in the timing of puberty in adult animals [31]. Placental specific protein 1(Plac1), was designated as candidate gene because Plac1was regarded as a marker for placental development [32], which was widely associated with prenatal growth and adult endocrine related disorders such as pubertal timing [29], obesity and diabetes [33]. Plac1 may control the timing of puberty by influencing the prenatal growth of mice.

On the other hand, clinical studies reported that variations of the X-chromosome in morphology and dosage were associated with abnormal pubertal development in humans. In 1983, Gardner reported that a white girl, having 46 chromosomes with a de novo reciprocal X:9 translocation, presented at the age of 16 with delayed puberty and primary amenorrhea [34]. Twenty years later, Talaban reported two brothers with hypogonadotropic hypogonadism (HH), obesity and short stature associated with a maternally inherited pericentric inversion (X) (p11.4q11.2) [35]. In other studies, different researchers independently reported that patients suffering from precocious or delayed puberty had abnormal X-chromosome dosage [36]–[40]. All of these investigations on the association between X-chromosome and pubertal disorder suggested chromosome X played regulatory roles in the pubertal timing. Comparative genomics designated nine major chromosome blocks of sequence homology between human and mouse X chromosomes [41]. Therefore it is inferred that chromosome X of mice also harbors gene(s) regulating the pubertal events.

It is important to note that the results of whole genome scanning are discrepant among three populations: direct, reciprocal and mixture population. The suggestive QTLs found in the three populations were not consistent, which could be attributed to limited scale of F2 population in our experiments and genes with minor contributive effects could not be exactly investigated. More importantly, because our previous study has suggested that the maternal nuclear genome contributes to the discrepancy of timing of puberty between C3H and B6 and many genes may regulate this trait by gene imprinting [16]. Some researchers have investigated that the maternal genotype can affect adult offspring phenotype including early growth, lipid, obesity, and diabetes in mice and argued that these genes may not identified by traditional analysis [42], [43]. The maternal effects are so obvious that the same or greater order of importance as the offspring's own genotype was reported in variation in offspring traits at multiple developmental stages [43], [44]. Therefore, the contribution of maternal genotype to offspring phenotype may result in the discrepancy in reciprocal crosses which leads to inconsistent QTL searching results. Imprinting genes analysis, together with intra-uterine physiological status study may help to provide important clues to the effects of maternal unclear genome on the timing of puberty in mice offspring.

However, for the identified QTL on chromosome X, analysis of the three populations is regarded as a feasible strategy to study the genetic pattern of the X-linked QTL and to avoid the biased result from single direction cross (see above). The QTL in our study was confirmed in a series of experiments. First, we made 10.000 permutation tests to confirm the threshold of LOD score and chose strict statistical criteria for a significant linkage based on genome-wide data. Then, the concordant results between linkage analysis and non-parametric test based on DXMit166 confirmed the possible QTLs on chromosome X. Moreover, the result from modified CSS and ISCSs further supported that the QTL was associated with the timing of puberty onset in mice.

In summary, the identification of a QTL on chromosome X regulating the timing of VO in mice represents a step further towards finding of novel factors regulating the onset of puberty. It also expands our knowledge that X chromosome plays an important role in pubertal development, in addition to its well recognized determination of sex. The further work on fine mapping of this chromosome region to identify associated gene(s) may help to uncover the underlying mechanism of pubertal timing in mammals.

## Materials and Methods

### Subject Animals, Housing and Care

All subjects were laboratory mice (*Mus musculus*) and all procedures were undertaken with the relevant ethical approvals. Male and female mice of the inbred strains C57BL/6J (B6) and C3H/HeJ (C3H) were obtained from Shanghai SLAC Laboratory Animal Co. LTD (Shanghai, China). Pedigrees in study were from three generations: two inbred strains, two F1 crosses (B6♀×C3H♂ [B6C3HF1], C3H♀×B6♂[C3HB6F1]) and two F2 crosses (B6C3HF1♀×B6C3HF1♂[B6C3HF2], C3HB6F1♀×C3HB6F1♂[C3HB6F2]). All mice used in this study were housed in standard polysulfone microisolator cages with hardwood chips (Shanghai SLAC Laboratory Animal CO. LTD) and were allowed unlimited access to water and food (Shanghai SLAC Laboratory Animal CO. LTD). For breeding, individual males were placed in cages each with 2–3 females until the female was obviously pregnant. The date of birth was designated as the day pups were observed. Pups were weaned between the age of 20 to 21 days, and males and females were then housed separately. No more than 5 female pups were housed per cage to ensure that access to food and water was unfettered. All animal housing and care procedures were conducted in accordance with the Experimental Animal Management Ordinance of P.R.China (1988).

### Assessment of Pubertal Timing

Beginning on the day of weaning, mice were examined daily from 8:00 a.m. to 11:00 a.m., and the date of VO for female and concurrent body weight were recorded. All females of the two inbred strains, two F1 crosses and two F2 crosses were studied for the age at VO. To ensure that no environmental or methodological changes occurred during the survey that might have systematically affected the results, the progenitor strains (B6 and C3H) were periodically rebred and the timing of VO was reassessed throughout the course of the experimentation to verify that the observed data of VO in F1 and F2 were persistent.

### Use of F2 Extremes to Identify Chromosomal Regions Containing the timing of VO Genes

Two sample groups, E (earlier-onset) and L (later-onset), were made by selecting phenotypically extreme individuals from F2 intercross population of 464 mice in the summer of 2005. Extreme progenies with the phenotype more than or less than 2 SD (standard deviation) from the mean in direct and reciprocal F2 population with litters ranging from 7–12 pups in size were selected. A total of 161 reciprocal and 99 direct F2 intercross female mice were selected.

### Construction of Modified ISCS for Fine mapping QTL on ChrX

Male F2 mice were genotyped and the individuals that held at least one recombination at the special interval (DXMit103∼DXMit119) were chosen and then backcrossed with female C3H to obtain N_2_ generation. The female N_2_ continued to backcross with male C3H to generate N_3_ generation. N_3_ male mice and individuals were genotyped and those held only one recombination at the target interval were selected and continued to backcross with female C3H to generate N_4_. At last, the age at VO of all female mice of N_4_ progenies was recorded. All modified ISCSs were verified by genotyping genetics markers on chromosome X (see figure 5). In the same manner, a modified chromosome substitution strain was also generated: C3H-X^B^X^C^ with single copy of B6 chromosome X substituted into the C3H background.

In spite of the incompleteness in ISCS and CSS generation, they were proved to be applicable due to the significant contribution of the dominant QTL studied in our research.

### DNA Extraction and Genotyping of STR and SNP Markers

After extracting DNA from the tail tip using a DNA Extraction Kit (BioTech, Beijing, China), 109 Short Tandem Repeats (STRs) and 4 Single Nucleotide Polymorphisms (SNPs) spaced at approximate 10–20 cM intervals throughout the genome were applied in whole genome scanning ( Table S1. See Supporting Information). Eleven STRs and four SNPs were also genotyped in construction of mISCSs and mCSS.

Fluorescence-PCR was carried out in the standard procedures. PCR products from amplification of F2, B6, and C3H mice were separated on 6% polyacrylamide gels using 377 sequencer (Applied Biosystems Incorporation, USA) and analyzed by Genemapper 3.2. Map positions in centimorgan were measured from centromere, as reported in the 2005 Mouse Genome Database (http://www.informatics.jax.org/). Typing of the selected single nucleotide polymorphisms (SNPs) was performed with a method developed by ligase detection reaction [45]. We reviewed the genotypes for the presence of double recombinants over short genetic distances, and questionable genotypings or missing data were repeated. Alleles from C57BL/6J strain were designated as “B6,” and alleles from C3H/HeJ as “C3H”.

### Data Analysis

To detect QTLs regulating the timing of VO, we analyzed three populations: one included extreme individuals only from the F2 direct cross population (B6C3HF2), one from their reciprocal counterparts (C3HB6F2) and the mixture population from grouping the two crosses.

Linkage analysis was performed using the package R/qtl based on R-program designed for mapping QTLs in the experimental crosses (http://www.biostat.jhsph.edu/kbroman/qtl/)). For the sake of saving time, the strategy of selective genotyping of the extreme progeny was applied in this experiment. When analyzing data, all phenotypes were considered for the F2 individuals but only the genotypes of individuals in the tails of the phenotypic distribution were selected; the genotypes for those in the middle were entered as missing [46]–[48]. A stratified permutation was applied in determining the threshold to avoid the possible biased thresholds due to only some of mice being genotyped in this study [49]. Also, considering the character of chromosome X, a specified permutation that can discriminate autosome and chromosome X was performed to derive a more liable threshold [50]. Among all analyses, empirical significance thresholds (p<0.63, p<0.05 and p<0.01, respectively) were determined through analysis of 10,000 permutations of the F2 genotypic and phenotypic data based on the package R/qtl.

As multiple microsatellites were tested and the selective chromosome linkage data were limited, the positive results generated from J/qtl were analyzed again using two-tailed, nonparametric tests for independent variables (Mann-Whitney U Tests). Mann-Whitney U Tests were applied because vaginal opening was probably not a normally distributed trait in mice. The data of genotype and phenotype among mISCS also analyzed using Mann-Whitney U Tests. Analyses were performed using Statistical Product and Service Solution (SPSS 13.0). Significance was attributed to p<0.05 for all tests.

## Supporting Information

Table S1 The 113 markers genotyped in this study
(0.09 MB DOC)

## References

[1] Kadlubar FF, Berkowitz GS, Delongehanp RR (2001). The putative high-activity variant, CYP3A4*1B, predicts the onset of puberty in young girls.. Proc Am Assoc Cancer Res.

[2] Kadlubar FF, Berkowitz GS, Delongchamp RR, Charles W, Green LB (2003). The CYP3A4*1B variant is related to the onset of puberty, a known risk factor for the development of breast cancer.. Cancer Epidemiol Biomarkers Prev.

[3] Roux N, Genin E, Carel JC, Matsuda F, Chaussain JL (2003). Hypogonadotropic hypogonadism due to loss of function of the KiSS1-derived peptide receptor GPR54.. Proc Natl Acad Sci USA.

[4] Seminara SB, Messager S, Chatzidaki EE, Thresher RR, Acierno JS (2003). The GPR54 gene as a regulator of puberty.. N Engl J Med.

[5] Palmert MR, Hischhorn JN (2003). Genetic approaches to stature, pubertal timing, and other complex traits.. Mol Genet Metab.

[6] Nelson JF, Felicio KK, Johnson TE (1990). Genetic influences on the timing of puberty in mice.. Biol Reprod.

[7] Nelson JF, Karelus K, Felicio LS, Johnson TE (1992). Genetic influences on oestrous cyclicity in mice: evidence that cycle length and frequency are differentially regulated.. J Reprod Fertil.

[8] Chehab FF, Mounzih K, Lu R, Lim ME (1997). Early onset of reproductive function in normal female mice treated with leptin.. Science.

[9] Krewson TD, Supelak PJ, Hill AE, Singer JB, Lander ES (2004). Chromosomes 6 and 13 harbor genes that regulate pubertal timing in mouse chromosome substitution strains.. Endocrinology.

[10] Nathan BM, Hodges CA, Supelak PJ, Burrage LC, Nadeau JH (2006). A Quantitative Trait Locus on Chromosome 6 Regulates the Onset of Puberty in Mice.. Endocrinology.

[11] Therman E, Laxova R, Susman B (1990). The critical region on the human Xq.. Human Genet.

[12] Geerkens C, Just W, Vogel W (1994). Deletions of Xq and growth deficit: a review.. Am J Med Genet.

[13] Maraschio P, Tupler R, Barbierato L, Dainotti E, Larizza D (1996). An analysis of Xq deletions.. Hum Genet.

[14] Carruth LL, Reisert I, Arnold AP (2002). Sex chromosome genes directly affect brain sexual differentiation.. Nat Neurosci.

[15] Sisk CL, Foster DL (2004). The neural basis of puberty and adolescence.. Nat Neurosci.

[16] Zhou YX, Zhu WS, Guo ZX, Zhao Y, Song ZJ (2007). Effects of maternal nuclear genome on the timing of puberty of offspring in mice.. J Endocrinol.

[17] Brockmann GA, Kratzsch J, Haley CS, Renne U, Schwerin M (2000). Single QTL effects, epistasis, and pleiotropy account for two-thirds of the phenotypic F-2 variance of growth and obesity in DIU6i DBA/2 mice.. Genome Res.

[18] Yang RC (2004). Epistasis of quantitative trait loci under different gene action models.. Genetics.

[19] Darvasi A (1997). Interval-specific congenic strains (ISCS): an experimental design for mapping a QTL into a 1-centimorgan interval.. Mamm Genome.

[20] Iakoubova OA, Olsson CL, Dains KM (2001). Genome-tagged mice (GTM): two sets of genome-wide congenic strains.. Genomics.

[21] Darvasi A, Soller M (1995). Advanced intercross lines, an experimental population for fine genetic mapping.. Genetics.

[22] Rogner UC, Avner P (2003). Congenic mice: cutting tools for complex immune disorders.. Nat Rev Immunol.

[23] Markel P, Shu P, Ebeling C, Carlson GA, Nagle DL (1997). Theoretical and empirical issues for marker-assisted breeding of congenic mouse strains.. Nat Genet.

[24] Kennedy GC, Mitra J (1963). Hypothalamic control of energy balance and the reproductivecycle in the rat.. J Physiol.

[25] Ishikawa A, Hatada S, Nagamine Y, Namikawa T (2005). Further mapping of quantitative trait loci for postnatal growth in an inter-sub-specific backcross of wild *Mus musculus castaneus* and C57BL/6J mice.. Genet Res.

[26] Ishikawa A, Matsuda Y, Namikawa T (2000). Detection of quantitative trait loci for body weight at 10 weeks from Philippine wild mice.. Mamm Genome.

[27] Ishikawa A, Namikawa T (2004). Mapping major quantitative trait loci for postnatal growth in an intersubspecific backross between C57BL/6J and Philippine wild mice by using principal component analysis.. Genes Genet Syst.

[28] Hemberger MC, Pearsall RS, Zechner U, Orth A, Otto S (1999). Genetic Dissection of X-Linked Interspecific Hybrid Placental Dysplasia in Congenic Mouse Strains.. Genetics.

[29] Ibanez L, Jimenez R, de Zegher F (2006). Early puberty-menarche after precocious pubarche: relation to prenatal growth.. Pediatrics.

[30] Peluso JJ, Pappalardo A, Losel R, Wehling M (2006). Progesterone membrane receptor component 1 expression in the immature rat ovary and its role in mediating progesterone's antiapoptotic action.. Endocrinology.

[31] Terasawa E, Fernandez DL (2001). Neurobiological mechanisms of the onset of puberty in primates.. Endocr Rev.

[32] Cocchia M, Huber R, Pantano S, Chen EY, Ma P, Forabosco A, Ko MS, Schlessinger D (2000). *PLAC1*, an Xq26 gene with placenta-specific expression.. Genomics.

[33] Wallace JM, Aitken RP, Milne JS, Hay WW (2004). Nutritionally mediated placental growth restriction in the growing adolescent: consequences for the fetus.. Biol Reprod.

[34] Gardner HA, McConnon JK, MacKenzie MA (1983). An X;9 translocation, primary amenorrhea, and hypothalamic dysfunction.. Am J Med Genet.

[35] Talaban R, Sellick GS, Spendlove HE, Howell R, King C (2005). Inherited pericentric inversion (X) (p11.4q11.2) associated with delayed puberty and obesity in two brothers.. Cytogenet Genome Res.

[36] Mark HF, Bayleran JK, Seifer DB, Meyers-Seifer CH (1996). A combined cytogenetic and molecular approach for diagnosing delayed puberty.. Clin Pediatr (Phila).

[37] Hockey A (1986). X-linked intellectual handicap and precocious puberty with obesity in carrier females.. Am J Med Genet.

[38] Devi AS, Metager DA, Luciano AA, Benn PA (1998). 45, X/46, XX mosaicism in patients with idiopathic premature ovarian failure.. Fertil Steril.

[39] Jarvela IE, Salo MK, Santavuori P, Salonen RK (1993). 46, XX/69,XXX diploid-triploid mixoploidy with hypothyroidism and precocious puberty.. J Med Genet.

[40] Grosso S, Berardi R, Pucci L, Balestri P (1999). Precocious puberty with diploid-triploid mixoploidy with hypothyroidism and precocious puberty.. J Med Genet.

[41] Ross MT, Grafham DV, Coffey AJ, Scherer S, Mclay K (2005). The DNA sequence of the human X chromosome.. Nature.

[42] Jarvis JP, Kenney-Hunt J, Ehrich TH, Pletscher LS, Semenkovich CF (2005). Maternal genotype affects adult offspring lipid, obesity, and diabetes phenotypes in LGXSM recombinant inbred strains.. Lipid Res.

[43] Wolf JB, Vaughn TT, Pletscher LS, Cheverud JM (2002). Contribution of maternal effect QTL to genetic architecture of early growth in mice.. Heredity.

[44] Cheverud JM, Moore AJ, Boake CRB (1994). Quantitative genetics and the role of the environment provided by relatives in the evolution of behavior.. Quantitative Genetic Studies of Behavioral Evolution.

[45] Xiao Z, Xiao J, Jiang Y, Zhang S, Yu M (2006). A novel method based on ligase detection reaction for low abundant YIDD mutants detection in Hepatitis B virus.. Hepatol Res.

[46] Rabbee N, Speca D, Armstrong NJ, Speed TP (2004). Power calculations for selective genotyping in QTL mapping in backcross mice.. Genet Res.

[47] Lander ES, Botstein D (1989). Mapping mendelian factors underlying quantitative traits using RFLP linkage maps.. Genetics.

[48] Haston CK, Wang M, Dejournett RE, Zhou X, Ni D (2002). Bleomycin hydrolase and a genetic locus within the MHC affect risk for pulmonary fibrosis in mice.. Hum Mol Genet.

[49] Manichaikul A, Palmer AA, Sen S, Broman KW (2007). Significance thresholds for quantitative trait locus mapping under selective genotyping.. Genetics.

[50] Broman KW, Sen S, Owens SE, Manichaikul A, Southard-Smith EM (2006). The X chromosome in quantitative trait locus mapping.. Genetics.

