# Supplementary material for: A Dominant X-Linked QTL Regulating Pubertal Timing in Mice Found by Whole Genome Scanning and Modified Interval-Specific Congenic Strain Analysis
Source: PLoS One. 2008 Aug 22;3(8):e3021. doi: 10.1371/journal.pone.0003021 (PMC2516528; doi:10.1371/journal.pone.0003021)
Supplement: Table S1 — The 113 markers genotyped in this study (0.09 MB DOC) [file pone.0003021.s001.doc]

**Table 1S**. The 113 markers genotyped in this study.

| Marker | Pos. | Marker | Pos. | Marker | Pos. | Marker | Pos. |
| --- | --- | --- | --- | --- | --- | --- | --- |
| rs3696408 | 7.1 | D6Mit204 | 4.4 | D11Mit227 | 2 | D16Mit129 | 3.4 |
| D1Mit233 | 23.6 | D6Mit268 | 15.6 | D11Mit271 | 21 | D16Mit101 | 17 |
| D1Mit23 | 41 | D6Mit274 | 20.5 | D11Mit29 | 40 | D16Mit157 | 34.1 |
| D1Mit386 | 59.5 | D6Mit243 | 30.4 | D11Mit124 | 57.8 | D16Mit151 | 45.5 |
| D1Mit102 | 73 | D6Mit261 | 37 | D11Mit104 | 79 | D16Mit106 | 71.5 |
| D1Mit36 | 92.3 | D6Mit39 | 46.3 |  |  |  |  |
| D1Mit210 | 109 | D6Mit150 | 51 | D12Mit264 | 1 | D17Mit171 | 5 |
|  |  | D6Mit254 | 60.6 | D12Mit60 | 16 | D17Mit104 | 21.7 |
| rs3695983 | 3.4 | D6Mit59 | 67 | D12Mit74 | 29 | D17Mit217 | 38.5 |
| D2Mit365 | 17 | D6Mit201 | 74.1 | D12Mit179 | 45 | D17Mit129 | 55.7 |
| D2Mit91 | 37 |  |  | D12Mit144 | 61 |  |  |
| D2Mit103 | 55.7 | D7Mit152 | 1 |  |  | D18Mit18 | 2 |
| D2Mit409 | 74.3 | D7Mit228 | 18 | D13Mit17 | 8 | D18Mit113 | 20 |
| D2Mit142 | 92 | D7Mit346 | 34 | D13Mit266 | 16 | D18Mit183 | 37 |
| D2Mit148 | 105 | D7Mit149 | 50 | D13Mit179 | 30 | D18Mit213 | 55 |
|  |  | D7Mit332 | 65.6 | D13Mit248 | 34 |  |  |
| D3Mit221 | 2.4 |  |  | D13Mit231 | 39 | D19Mit42 | 5 |
| D3Mit225 | 22 | D8Mit124 | 6 | D13Mit147 | 49 | D19Mit63 | 24 |
| D3Mit357 | 44.8 | rs13479709 | 22 | D13Mit148 | 59 | D19Mit90 | 41 |
| D3Mit110 | 64.1 | D8Mit283 | 38.6 | D13Mit262 | 68 | D19Mit137 | 55.7 |
| D3Mit162 | 84.9 | D8Mit114 | 53 |  |  |  |  |
|  |  | D8Mit56 | 73 | D14Mit11 | 0.7 | DXMit103 | 4.2 |
| D4Mit101 | 3.2 |  |  | D14Mit59 | 15 | DXMit166 | 15.5 |
| D4Mit163 | 21.9 | D9Mit192 | 26 | D14Mit143 | 28.4 | DXMit140 | 19 |
| D4Mit301 | 42.5 | D9Mit166 | 41 | D14Mit263 | 44.4 | DXMit119 | 29.5 |
| D4Mit71 | 61.9 | D9Mit184 | 60 | D14Mit77 | 60 | DXMit16 | 37 |
| D4Mit42 | 81 | D9Mit82 | 74 |  |  | DXMit172 | 48.7 |
|  |  |  |  | D15Mit102 | 6.7 | DXMit130 | 55 |
| D5Mit344 | 1 | D10Mit298 | 3 | D15Mit100 | 20.2 | DXMit10 | 63.2 |
| D5Mit75 | 20 | D10Mit214 | 19 | D15Mit92 | 35.3 | DXMit223 | 73.3 |
| D5Mit308 | 44 | D10Mit31 | 36 | D15Mit159 | 49.6 |  |  |
| D5Mit367 | 65 | D10Mit231 | 52 | D15Mit79 | 66.2 |  |  |
| D5Mit287 | 86 | D10Mit271 | 70 |  |  |  |  |

Pos., map positions in centimorgan were measured from centromere, as reported in the 2005 Mouse Genome Database (http://www.informatics.jax.org/).
